# Lipoma of the cheek presenting with recurrent sialadenitis of the right parotid gland: a case report

**DOI:** 10.1186/s13256-016-1099-9

**Published:** 2016-11-03

**Authors:** Osundwa Tom, Ochola Tom

**Affiliations:** Department of Oral and Maxillofacial Surgery, Oral Pathology/Medicine and Oral Radiology, University of Nairobi Dental Hospital, P.O. Box 19676-00202, Nairobi, Kenya

**Keywords:** Lipoma, Sialadenitis, Case report, Recurrent, Cheek and mucosa

## Abstract

**Background:**

Lipomas are benign neoplasms arising from adipose tissue. Oral lipomas have been reported in the buccal mucosa, tongue, floor of the mouth and lips; however, the case of a lipoma occurring as an antecedent lesion to recurrent sialadenitis is hitherto unreported in the English literature.

**Case presentation:**

We report the case of an intraoral lipoma occurring with signs and symptoms of recurrent sialadenitis in a 15-year-old Kenyan girl of Kikuyu descent. The lipoma was antecedent leading to partial obstruction and stasis related to the right Stensen’s duct culminating in recurrent sialadenitis of the ipsilateral parotid gland. Due to the slow growth, softness, diffuse nature and lack of pain, lipomas may exist below the diagnostic radar, hence, the need to have a high index of suspicion and utilize diagnostic aids as necessary. In this case magnetic resonance imaging was key in establishing the existence of the lipoma. The lipoma was excised with resolution of the recurrent sialadenitis.

**Conclusions:**

The purpose of this report is to present the diagnostic challenge emanating from the pressure effects of an intraoral soft tissue lipoma masquerading as recurrent sialadenitis with a view to improving on patient care through sensitization.

## Background

Lipomas are benign neoplasms arising from adipose tissue. Though common elsewhere, lipomas are an uncommon neoplasm in the oral cavity. Lipomas stand out from normal body fat because their lipids are not subject to metabolism that normal body fat undergoes; in addition, they undergo uncontrolled proliferation. Intraoral lipomas are soft, slow-growing, mobile, and painless lesions that have been reported in the buccal mucosa, tongue, floor of the mouth, and lips [1, 2]. Intraoral lipomas are rare, with a prevalence rate of 1–4 % of all oral lesions [3]. Surgical excision is the preferred treatment and is rarely associated with recurrences [4]. We present the case of a patient with a cheek lipoma masquerading as recurrent sialadenitis of the right parotid gland, a rare occurrence hitherto unreported in the English literature.

## Case presentation

This is the case of a 15-year-old Kenyan girl of Kikuyu descent who presented with a diffuse, painful, slight cheek swelling on the right side of her face. The pain and swelling consistently increased in size just before and during meals. The painful area was well defined and the pain confined with no radiation. Her medical and dental histories were unremarkable except for treatment for otitis media 3 months before her presentation.

On examination her chronologic age was commensurate with her physique. The right parotid area was tender with no obvious change in the skin color. Intraorally, she had unerupted 8 s, missing 12, and a peg lateral in place of 22. The intraoral soft tissue was normal in color, texture, and consistency except around the right Stensen’s duct opening, which was inflamed. A small amount of pus was expressed from the right duct when slight pressure was applied on the papilla.

A diagnosis of acute suppurative sialadenitis was made and treatment executed in the form of copious fluid intake, amoxicillin and clavulanic acid (500 mg/12 5 mg) twice a day for 5 days, paracetamol 1000 mg three times a day for 5 days and povidone-iodine (Betadine) gargle for 7 days. The infection resolved completely until about a year later when she presented with signs and symptoms as those initially observed. A similar treatment regimen was prescribed and, after elimination of the infection, the patency of the right Stensen’s duct was checked by cannulation with no indication of obstruction.

**Fig. 1:**
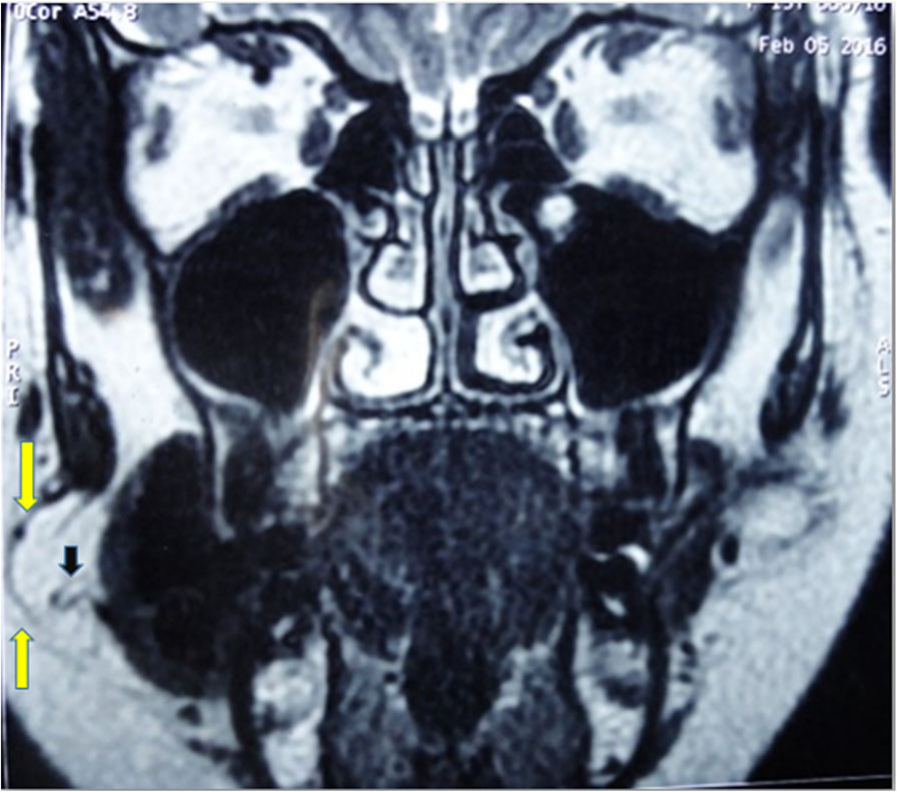
T2-weighted magnetic resonance image showing a well-defined right cheek lesion (*yellow arrows*). *Black arrow* points to the Stensen’s duct

**Fig. 2:**
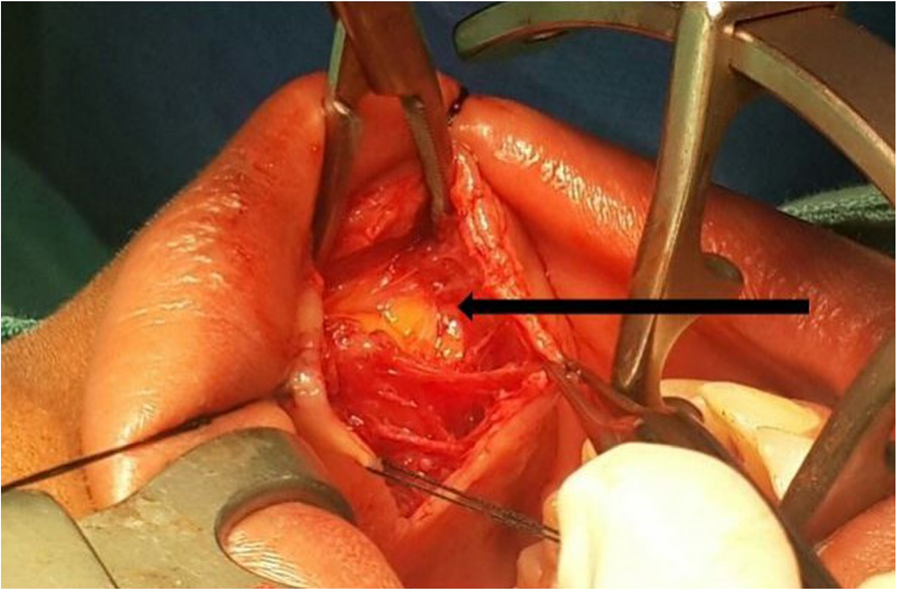
Incision on the right buccal mucosa to expose the lesion (*black arrow*)

**Fig. 3:**
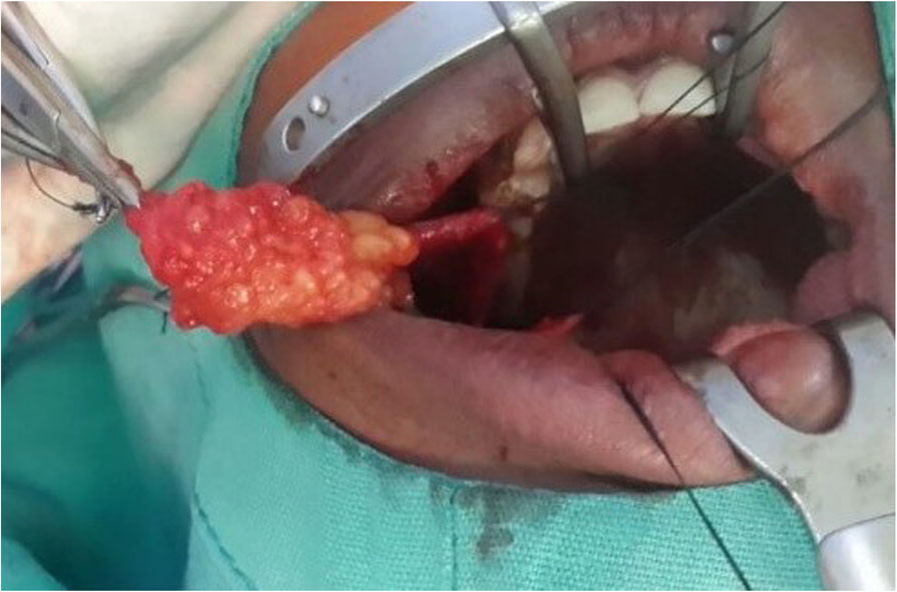
Lesion excised in total from the right intraoral buccal cheek area

About 2 years following her initial submission she presented with a recurrence of the initial signs and symptoms. It was immediately decided to perform a magnetic resonance imaging (MRI) scan (Fig. 1). This showed a homogenous well-defined right cheek lesion medial to the buccinator muscle and engulfing the ipsilateral Stensen’s duct. The clinical and radiographic features of the lesion were suggestive of a lipoma with the differential diagnosis of oral dermoid cyst, epidermoid cyst, and lymphoepithelial cysts considered. A decision was made to excise the lesion via an intraoral approach (Figs. 2 and 3). The Stensen’s duct was cannulated for localization and protection during the surgery. Following excision of the lesion, histopathology diagnosis confirmed a lipoma of the right cheek area. Immediately following recovery from the surgery, our patient reported complete resolution of previously noted symptoms of pain, discomfort, and swelling that were related to mealtimes. Six months following surgery, our patient is symptom-free and continues to be monitored.

## Discussion

The case presented is rare in the way the lipoma caused obstruction of the right Stensen’s duct resulting in recurrent infections of the ipsilateral parotid gland as the presenting pathology. This phenomenon stands hitherto undocumented in the English literature. Any attempts to treat the parotid gland in isolation would have been futile. Imaging in the form of an MRI scan in combination with the clinical findings resulted in the appropriate diagnosis and management of the condition. The location of the lesion, medial to the buccinator with almost no obvious external swelling while impinging on the Stensen’s duct, permitted the lesion to masquerade as sialadenitis of the right parotid gland and thus avoid immediate detection. It is therefore imperative to rule out external causes of stasis or blockage of the Stensen’s duct in the management of recurrent sialadenitis. It may be sometimes difficult to discern the lipoma from the surrounding soft tissue; hence, imaging is an important diagnostic tool when a lipoma is suspected. Some of the useful imaging modalities include ultrasound, computed tomography, and MRI. The intraoral approach guaranteed no external scarring and reduced the probability of injury to the facial nerve [5].

## Conclusions

The possibility of ductal obstruction by soft tissue lesions like lipomas is an important consideration in the management of sialadenitis. A high index of suspicion is required in making such a diagnosis, with the correct diagnostic imaging leading to more comprehensive patient care.
